# Tracer Study of St. Paul's Hospital Millennium Medical College Radiology Graduates: Career and Curriculum Insights

**DOI:** 10.4314/ejhs.v34i1.3S

**Published:** 2024-10

**Authors:** Alemayehu Bedane, Kumlachew Abate Mekonen, Ashenafi Aberra Buser, Tesfaye Kebede, Shimels Hussien Mohammed

**Affiliations:** 1 Department of Radiology and Medical Radiologic Technology, School of Medicine, St. Paul's Hospital Millennium Medical College, Addis Ababa, Ethiopia; 2 Department of Radiology, School of Medicine, Addis Ababa University, Addis Ababa, Ethiopia; 3 Department of Public Health, School of Public Health, St. Paul's Hospital Millennium Medical College, Addis Ababa, Ethiopia

**Keywords:** Radiology Education, Graduate Tracing, Career Satisfaction, Curriculum Study

## Abstract

**Background:**

Saint Paul's Hospital Millennium Medical College (SPHMMC) is one of Ethiopia's premier radiology training institutions. This study aimed to trace graduates of SPHMMC's radiology program and examined their career outcomes and perspectives on the training received.

**Methods:**

A cross-sectional study design was employed, recruiting 78 participants. Data on employment status, career outcomes, job satisfaction, and perceptions regarding the program's strengths, weaknesses, and curriculum relevance were collected through a web-based questionnaire from May 31 to June 8, 2024. SPSS version 26 was used for data processing and analysis.

**Results:**

The study revealed a high employment rate among SPHMMC radiology graduates, with 97% employed and 75% securing jobs within one month of graduation. Most graduates held permanent positions (86%) as radiology specialists, sub-specialists, and academicians. There was notable regional variation, with 65% working in Addis Ababa. Job satisfaction levels were 79% for radiology careers and 53% for current jobs. Satisfaction with the program overall and curriculum relevance was 69% and 96%, respectively.

**Conclusions:**

High employment rates, and career and curriculum satisfaction levels might be reflective of the high market demand for radiologists in Ethiopia and the effectiveness of SPHMMC's radiology program in preparing graduates for professional careers. The regional disparity in radiologist distribution suggests underlying systemic issues that require further investigation.

## Introduction

Saint Paul's Hospital Millennium Medical College (SPHMMC) is a specialized teaching hospital located in Addis Ababa, Ethiopia. The college provides tertiary medical interventions and trains health professionals across various fields, including medicine, nursing, and public health. Established in 2014, the radiology residency program at SPHMMC has emerged as one of Ethiopia's leading higher education institutes for radiology specialty and sub-specialty training. To date, the program has graduated over 110 radiologists who contribute significantly to the field both locally and internationally. The program aims to equip trainees with contemporary knowledge and skills in imaging technologies, including computed tomography (CT), ultrasound (US), magnetic resonance imaging (MPJ), and X-rays.

Graduate tracer studies not only track the status of alumni but also offer insights into the effectiveness of training programs. They explore essential outcomes such as employment status, employability levels, and job satisfaction, thereby indicating the societal relevance of various professions. In radiology, such studies can provide evidence regarding market demand, essential skills for professional practice, and the effectiveness of training programs in preparing graduates for successful careers.

To the best of our knowledge, this is the first tracer study conducted on SPHMMC radiology program graduates, and the findings will contribute to understanding their employment outcomes, career paths, and perceptions of curriculum relevance. This study is part of a comprehensive review in preparation for the radiology program's 10th-anniversary celebration in October 2024.

## Materials and Methods

**Study setting and participants**: The study took place at SPHMMC in Addis Ababa, Ethiopia, focusing on radiologists who completed their specialty training at the institution and were reachable through the alumni network. As of June 2024, SPHMMC had trained and graduated a total of 110 radiologists. After excluding deceased graduates and those without internet access, 90 alumni were estimated to be reachable and eligible for this study.

**Study design, sample size, and sampling approach**: This cross-sectional study included 90 radiologists, with a sample size calculated using a one-proportion formula adjusted for a small source population (N=90). The assumptions included a confidence level of 95%, a margin of error of 5%, and a job satisfaction level (p) of 41.17%. The required minimum sample size was determined to be 73 individuals, but given the small population, all eligible individuals were invited to participate.

### Variables and measurement

**Sociodemographic**: Sex, age, residence country, workplace, graduation year, job title, further Study

**Employment outcomes**: Employment Status, time to employment, means of employment, job contract, number of affiliations, monthly income, work sector, employer, job satisfaction, positions held,

### Leadership roles

**Curriculum views**: Curriculum satisfaction, curriculum relevance, education-job matching.

**Data collection**: Data were collected via a web-based questionnaire from May 31 to June 8, 2024. The questionnaire was distributed through email, Telegram, and WhatsApp groups, with multiple reminders sent to enhance participation. Most questions utilized a 5-point Likert scale for responses.

**Statistical analysis**: Data were analyzed using SPSS version 26, employing descriptive statistics to summarize the findings. Job satisfaction levels were categorized based on Likert scale responses.

**Ethical considerations**: The study protocol was reviewed and approved by the Institutional Review Board of SPHMMC. Participation was voluntary, and no personal identifying data were collected.

## Results

Sociodemographic characteristics: A total of 78 among 90 SPHMMC radiology graduates participated, resulting in a 86.7% response rate. The majority of respondents were male (74%), with a mean age of 34 years (SD=3 years). Most participants resided in Ethiopia (94%).

**Employment outcomes**: The employment outcomes showed that 97% of graduates were employed, with 75% securing jobs within one month. Recommendation through networks was the primary method for finding employment. Most graduates (86%) held permanent positions, predominantly in academia and hospital settings.

**Geographical distribution**: The majority of graduates (64%) worked in Addis Ababa, followed by Oromia (12%), Sidama (6%), and Harari (4%).

**Curriculum effectiveness and relevance**: Most graduates expressed high satisfaction with their career (79%) and the curriculum relevance (96%). Overall satisfaction with the program was 69%.

## Discussion

This graduate tracer study was conducted as part of a project to review SPHMMC radiology program status for its 10^th^ year anniversary. The review was multi-dimensional and involved various individuals and groups with a stake in the college's radiology program. This graduates tracer study specifically explored the views of the college's radiology alumni about their employment outcomes, job and career satisfaction, and views on the program's curriculum relevance, overall quality, and effectiveness in preparing students for professional roles.

We found high employment outcomes among SPHMMC radiology graduates. Almost all were employed, held top professional positions and found jobs immediately after graduation. This finding is consistent with previous reports that showed high levels of employability among medical professionals in Ethiopia compared to other fields (1, 10, 11). Thus, the high radiologist employment level found by this study was expected given the high demand for medical professionals and there were few radiologists in the country (1, 10, 12). The high employment rate indicates the limited number of radiologists in the country as well as the need to train further more radiologists to meet the needs and quality of medical services in the country. The main means of findings job was recommendation. This implies that it stands worthy of strengthening the network of SPHMMC radiology residents, fellows and graduates among themselves as well as with other national and international radiology communities. To this end, SPHMMC could contribute by establishing alumni websites, projects and professional engagements.

The high regional discrepancy in the distribution SPHMMC radiology graduates was concerning but might be due the nature of the profession and also underlying systematic differences in the regions. The concentration of over two-third of SPHMMC radiology graduates in just only Addis Ababa could highlight the urban-centric and tech-based nature of radiology practice. This is consistent with previous reports which showed major cities to be main working hubs for physicians (1, 10, 13). In urban areas in Ethiopia, there are better job opportunities, advanced medical facilities, high demand for imaging services, and a significant involvement the private sector in providing radiological services and better incentives for radiology professionals. The sparce concentration of radiologists in the other regions (Oromia, Amhara, Afar, Tigray, Gambela, Somali, Benishangul-Gumuz etc.) could also be related to the poor security situation of regions.

The high employment outcomes, career satisfaction and matching of education level and professional job among the SPHMMC radiology graduates was in agreement with the findings of other studies done in Ethiopia that reported a relatively higher professional career satisfaction among health professionals (9, 12, 13). Given almost all graduates (96%) indicated a high satisfaction with their curriculum relevance, the high employment rates and career satisfactions found by this study could also indicative of the alignment of the SPHMMC radiology training curriculum with real-world skill requirements and the overall effectiveness of the program in equipping graduates with the latest radiology knowledge and skills required by the industry (1, 11, 15). Compared to other domains, satisfaction with current job (53%) was relatively lower and might be taken an area of concern. Participants attributed this relatively lower job satisfaction level to the poor socioeconomic conditions of the country, impacting work setup, technical availability, living status and professional earnings. The finding of this study that a fifth of graduates reported their job to be a level up from their education might be taken as an indicator of a 20% further training in radiology sub-specialty fields. However, reader shouldn't take this finding as a conclusive and representative one because the study was limited to one institution and training needs change from time to time due to various evolving contexts.

The findings of this study have important implications for policy, research and educational practice. The high and favorable employment outcomes indicate the high market demand for radiologists and the need to open more radiology training programs and schools to ensure an adequate and equitable distribution of radiologists across Ethiopia (1,10). The concentration of graduates in Addis Ababa suggests a need for policies that incentivize work in areas far from the center. This might include availing better financial and professional incentive packages, decentralizing radiology services, and improving the security, living condition and infrastructure of undeserved regions. The findings of the study could serve as input in the revision of the SPHMMC radiology program curriculum. Academic curriculums need to address the needs and expectations of students. Further studies need to be done to explore the long-term career trajectories of SPHMMC radiology graduates. SPHMMC could contribute to this end by establishing an alumni cohort study. To ensure the SPHMMC radiology curriculum remains aligned with contemporary professional demands and research practices, it is imperative to incorporate recent technological advancements. Specifically, this entails integrating artificial intelligence, machine learning, specialized radiology research training, big-data analytics, and advanced research methodologies. To achieve this objective, SPHMMC could implement a comprehensive strategy encompassing continuous education programs, workshops, on-the-job and off-the-job training initiatives.

The study has some strengths and limitations worth mentioning. It aimed to comprehensively assess and gain valuable insights into various aspects of the SPHMMC radiology program and its graduates. To the best of the authors knowledge, this was the first radiology graduate's tracer study done in Ethiopia and it could serve as a model for other programs to review and improve their programs. The limitations of the study include a small sample size and a focus on a single institution, which would restrict the generalizability of the findings to national level. Additionally, the use of self-reported data about past experiences might have introduced biases such as recall bias and past positivity bias, where respondents tend to remember the past more positively than it might have been (16).

In conclusion, this study found favorable employment outcomes, as well as career and curriculum satisfaction, among SPHMMC radiology graduates. These outcomes likely reflect positively on the effectiveness of the college's radiology program in preparing trainees for real-world professional practice and the high market demand for radiologists in Ethiopia. However, the significant regional disparities in the availability of radiologists warrant attention to ensure equitable medical care across the country. SPHMMC needs to prioritize integrating artificial intelligence, machine learning, specialized radiology research training, and big data analytics into its curriculum to equip graduates for evolving professional demands and research advancements. A comprehensive continuous education strategy will further enhance faculty and student capabilities.

## Figures and Tables

**Table 1:** Sociodemographic characteristics of study participants (N=78)

Variables	Category	Number	%
Sex	Male	58	26
	Female	20	74
Age (years)	<=30	8	10
	31-34	43	55
	35-39	23	30
	>=40	4	5
Residence	Ethiopia	73	94
	Outside Ethiopia	5	6
Graduation	2017	4	5
Year	2018	10	13
	2019	10	13
	2020	4	5
	2021	25	32
	2022	4	5
	2023	19	24
	2024	2	3
Further	Yes	20	26
Education	No	58	74
Further	Neuroradiology	4	21
Study Fields	Body imaging	4	21
	Breast/women's imaging	3	16
	Cardiothoracic imaging	2	11
	Interventional radiology	2	11
	Public Health	2	11
	Management	1	5
	Theology	1	5

**Table 2:** SPHMMC radiology graduates' employment outcomes

Variables	Category	Number	%
Employment	Employed	76	97
	Not employed	2	3
Time taken to get job	<1 months	56	75
	1-6 months	18	24
	1-2 years	1	1
Job finding means	Recommendation	46	61
	Other means	14	19
	As walk-in applicant	8	11
	Arranged by school	5	7
	Advertisement	2	3
Job contracts	Permanent	65	86
	Part-time	18	24
Job titles	Radiology specialist	47	61
	Assistant professor	23	30
	Radiology fellow	4	5
	Consultant radiologist	2	3
	Lecturer	1	1
Affiliations	One	26	35
	Two	35	47
	Three	13	17
	Four	1	1
Monthly income	<80,000 Birr	9	27
	80,000-110,000 Birr	17	52
	>110,000 Birr	7	21
Return to sponsoring Organizations	Yes	48	64
	No	27	36
Job sector	Academic	37	49
	Hospital/clinic	35	46
	Diagnostic center	4	5
Employer	Government	50	66
	Private	41	54
Leadership	Yes	27	36
	No	49	64
Job satisfaction	Satisfied	40	53
	Average	29	38
	Dissatisfied	7	9

**Figure 1:**
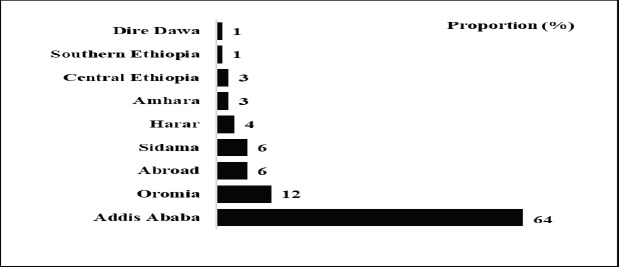
Geographical distribution of SHMMC alumni by Regions

**Figure 2:**
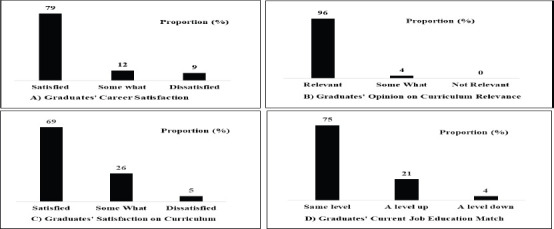
Graduates' opinions on the curriculum's career satisfaction relevance
